# Inflated pyroclasts in proximal fallout deposits reveal abrupt transitions in eruption behaviour

**DOI:** 10.1038/s41467-022-30501-6

**Published:** 2022-05-20

**Authors:** Thomas J. Jones, Yannick Le Moigne, James K. Russell, Glyn Williams-Jones, Daniele Giordano, Donald B. Dingwell

**Affiliations:** 1 Department of Earth, Ocean and Ecological Sciences, University of Liverpool, Liverpool, L69 3GP UK; 2 Centre for Natural Hazards Research, Department of Earth Sciences, Simon Fraser University, Burnaby, BC Canada; 3 Laboratoire Magmas et Volcans, Université Clermont-Auvergne, Clermont-Ferrand, France; 4 Department of Earth, Ocean and Atmospheric Sciences, University of British Columbia, Vancouver, BC Canada; 5 Dipartimento di Scienze della Terra, Universitá degli studi di Torino, 10125 Torino, Italy; 6 Department of Earth and Environmental Sciences, Ludwig-Maximilians-Universität, 80333 Munich, Germany

**Keywords:** Natural hazards, Volcanology

## Abstract

During explosive eruption of low viscosity magmas, pyroclasts are cooled predominantly by forced convection. Depending on the cooling efficiency relative to other timescales, a spectrum of deposits can be formed. Deposition of hot clasts, above their glass transition temperature, can form spatter mounds, ramparts and clastogenic lava flows. Clasts may also be deposited cold, producing tephra cones and blankets. Thus, the deposit and pyroclast type can provide information about eruption dynamics and magma properties. Here we examine pyroclasts from Tseax volcano, British Columbia, Canada. These newly identified inflated pyroclasts, are fluidal in form, have undergone post-depositional expansion, and are found juxtaposed with scoria. Detailed field, chemical and textural observations, coupled with high temperature rheometry and thermal modelling, reveal that abrupt transitions in eruptive behaviour — from lava fountaining to low-energy bubble bursts — created these pyroclastic deposits. These findings should help identify transitions in eruptive behaviour at other mafic volcanoes worldwide.

## Abstract (web summary)

Jones et al. identify a new type of volcanic deposit, inflated pyroclasts, that form by continued gas expansion post-deposition. They show how these deposits can be indicative of abrupt transitions in eruptive behaviour at mafic volcanoes.

## Introduction

Low viscosity magmas are the most common erupted on Earth^1,2^. The associated eruptions can be long-lived, causing large economic losses and can be highly variable both in magnitude and style, thus, making hazard management challenging^3–6^. The style and efficiency of magma fragmentation directly controls the type of products erupted and, in turn, the hazards they pose^7–9^. For example, Hawaiian style fountaining of fluidal clasts frequently produces vent proximal spatter ramparts and mounds but can also yield coeval lava flows where accumulation rates are high enough and clasts remain hot^10–14^ for a longer time. Such lavas pose an additional risk to communities and infrastructure down flank^3^. If fragmentation is highly efficient and clasts are cooled to a greater extent, the resulting widespread tephra blankets pose a risk to a larger geographic area and in some cases may even cause closure of airspace^15^. Despite the diverse array of hazards that such mafic eruptions pose, too little is known about pyroclast transport and the associated eruption dynamics^7,16,17^ in such eruptions.

Explosive activity at mafic volcanoes, especially in its milder forms (i.e., lava fountaining, spattering events and discrete bubble bursts) can be highly variable in its surface expression^18–20^. Eruption intensities and styles of activity can also fluctuate over short timescales and distances^21,22^—this presents challenges to hazard management^23^ and hinders systematic observation of eruptive phenomena. Fluctuations in intensity and style can occur between eruptive episodes, within a single episode, and simultaneously at different spatial locations^21^. Furthermore, the physical processes governing explosive eruption of low viscosity magmas are fundamentally different to those operating in the now relatively well-studied silicic systems^24–27^. Most commonly their fragmentation processes are fluid dynamic in nature such that breakage does not occur simply by decompression upon crossing the glass transition^7,27–29^. Instead, their low melt viscosities allow surface tension-driven reshaping and bubble nucleation, growth, and coalescence to operate on syn- and post-eruptive timescales. A common example includes achneliths such as Pele’s tears where in-flight surface tension-driven relaxation^30^ transforms irregular pyroclast morphologies into more spherical shapes. The consequence is that pyroclast properties are susceptible to modification after the initial fragmentation^12,29–33^. As a result, the eruptive deposits derived from low viscosity magmas are challenging to interpret as: (1) vent structures evolve and migrate during an eruption;^34–36^ (2) early products are frequently buried or rafted by subsequent eruption episodes^37–39^ and (3) rheomorphic processes and in-flight secondary fragmentation processes destroy primary fragmentation features^1,11,17,29,33,40,41^. These multiple factors hinder our understanding of mafic eruption dynamics solely by viewing their associated products.

Here we examine pyroclasts from the ~1700s CE eruption of Tseax in northwest British Columbia, Canada^42–45^, which is reported to have caused up to 2,000 fatalities making it the deadliest eruption in Canada’s history^46,47^. The pyroclasts are fluidal in form and have undergone post-depositional expansion and are therefore termed inflated pyroclasts herein. We performed a set of detailed field, chemical, and textural observations coupled with high temperature rheometry and thermal modelling to reveal the unique and abrupt transitions in eruptive behaviour that created these pyroclasts.

## Results and discussion

### Tseax volcano

**Fig. 1:**
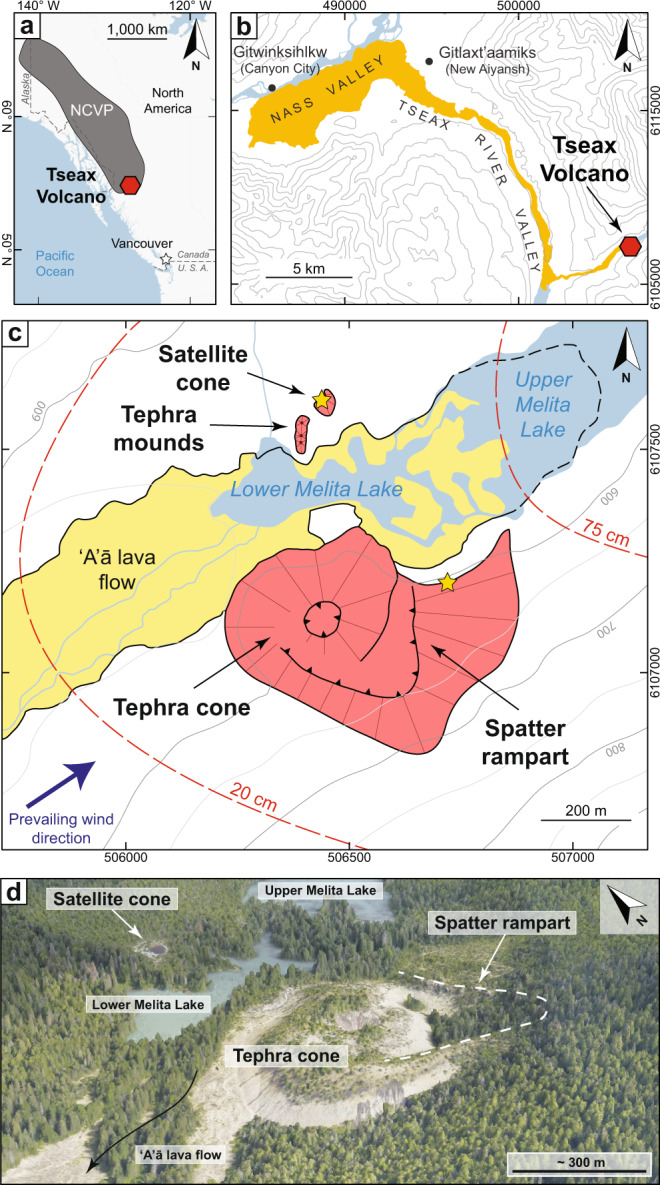
Location and description of the area surrounding Tseax volcano. **a** Tseax (red hexagon) in the context of Neogene-Quaternary volcanic centres and complexes of the Northern Cordilleran Volcanic Province (NCVP). **b** Map of Tseax and the 32-km-long valley-filling lava flows (yellow). **c** Volcanological map of Tseax cone, the Satellite cones and spatter rampart, all shaded in red. Yellow stars indicate locations of inflated pyroclast samples. Red contours denote tephra deposit thickness. Modified after Le Moigne et al.^44^. **d** Oblique 3D view of Tseax and surrounding area generated by digital photogrammetry. Modified after Le Moigne et al.^44^.

Tseax volcano, located in the Nisga’a Memorial Lava Bed Provincial Park in northwest British Columbia, Canada, is the southernmost volcanic centre of the Northern Cordillera Volcanic Province^48^ and is notable for a 32-km-long basanite-trachybasalt lava flow (~0.5 km^3^ covering ~36 km^2^; Fig. 1). The tephra cone and valley-filling lavas overlay intercalated sandstone to siltstone and mudstone from the late Jurassic Bowser Lake group^49,50^. Detailed volcanological mapping shows that Tseax volcano consists of a ~65 m high, 350-400 m diameter tephra cone (2.8 ± 0.4 × 10^6^ m^3^) situated within a 550-600 m diameter oxidised horseshoe-shaped spatter rampart^44^. Another smaller (~20 m high, ~55 m diameter, 1.7 ± 0.1 ×10^4^ m^3^) highly oxidised tephra cone, unnamed but referred to here as Satellite cone, is located 470 m to the north of Tseax and in close proximity to a group of small tephra mounds^44^ (Fig. 1).

### Deposit description

**Fig. 2:**
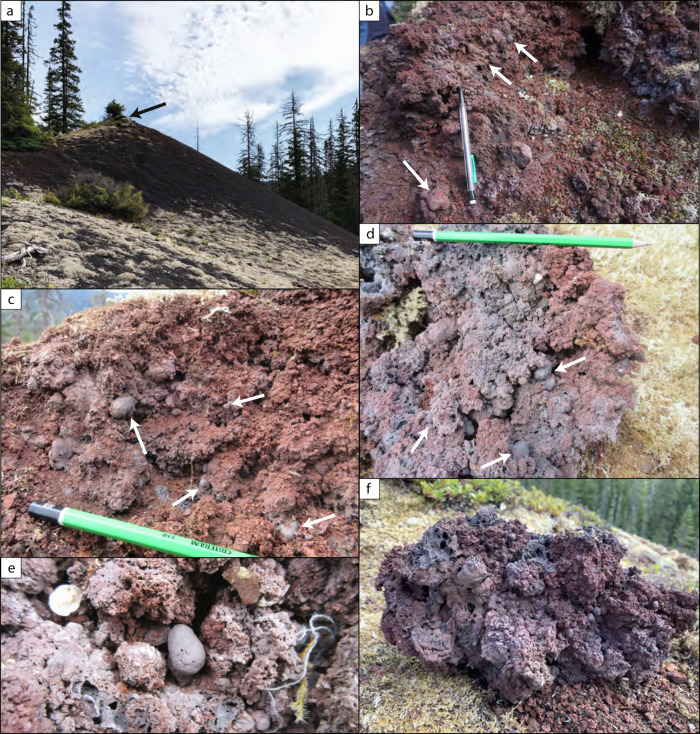
Deposit scale observations of the Tseax Satellite cone. **a** Satellite scoria cone (height ~20 m) with inflated pyroclasts only found within ~5 m of the summit. The black arrow points to the location of the other figure panels. **b**–**d** Variably oxidised scoria cone deposits at the summit in the surface deposits. Some inflated pyroclasts are identified by the white arrows. These clasts have a smooth outer surface, a fluidal morphology and are commonly rounded in shape. **e** Close up of an inflated pyroclast (~5 cm) surrounded by scoria. **f** Close up of a dissected block (30 cm in length) found at the summit of the Satellite cone which contains several inflated pyroclasts that are fluidal in nature and infill the space between scoria clasts. Further deposit photographs can be found in Fig. S1.

The highly vesicular, fluidal inflated pyroclasts were found at two separate vent proximal locations at Tseax volcano (yellow stars in Fig. 1c). The southern site is a spatter rampart wall, containing rare inflated pyroclasts and slightly rheomorphic spatter clasts that have been variably oxidised. At this location, the inflated pyroclasts make up <2% of the deposit by volume and are not concentrated in any stratigraphic horizon. The northern site features much better exposure and is the primary location sampled and investigated in this study. The inflated pyroclasts occur within a ~5 m radius of the top of the Satellite cone (Fig. 2a). The near-summit region of the Satellite cone has no pit or crater. The deposit is variably oxidised and dominated by moderately to well-sorted scoria clasts, with the inflated pyroclasts contributing <5% of the deposit by volume. No clear deposit stratigraphy was identified. In places the deposit is agglutinated, however individual pyroclasts can be identified (Fig. 2b**–**d) and in most cases the inflated pyroclasts can be separated from the deposit by hand whilst remaining intact. Although partially agglutinated, there is no evidence of compactional welding at the Satellite cone. The inflated pyroclasts are only observed at vent proximal locations and fill the available void space in the deposit (Fig. 2e, f). In some cases, they are observed to have drained by gravity within the deposit or very occasionally are deformed by the pyroclast(s) directly above, forming squeeze-out textures. Further deposit photographs can be found in Fig. S1.

### Inflated pyroclast morphology and surfaces

**Fig. 3:**
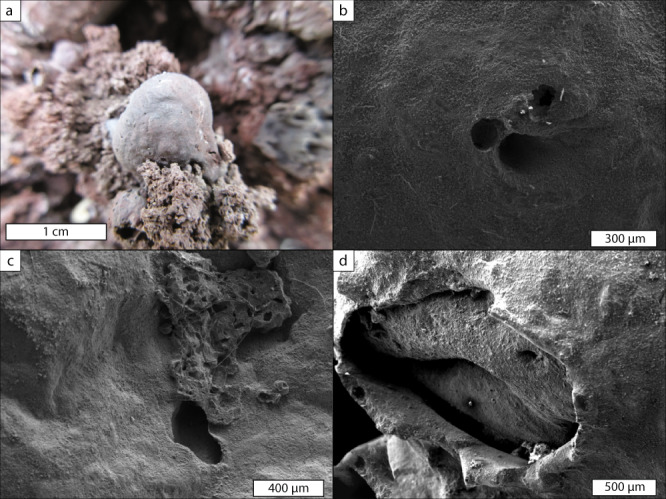
Inflated pyroclast textures and detailed features. **a** Close up photograph of an inflated pyroclast within the scoria cone deposit. The clast surface contains a few small ($\lesssim$0.5 mm) holes. **b**–**d** Scanning electron micrographs of inflated pyroclast surfaces showing: **b** circular non-brittle holes on the pyroclasts surface; **c** smaller pieces of vesicular scoria stuck to the inflated pyroclast and **d** multiple layers or skins. Further photographs of individual pyroclasts can be found in Fig. S2.

The individual inflated pyroclasts are commonly spherical in shape (Fig. 3a), but more elongated morphologies are also found. Their surfaces have a dull, matt appearance, rather than the shiny, glassy surfaces commonly observed on Pele’s tears, for example. We interpret this dull appearance to be a consequence of microlitic^51^ and possibly further nanolitic^52^ crystallisation (see next sub-section). The surfaces of the inflated pyroclasts are pierced by a few randomly distributed (sub)circular holes that are on the order of 100 μm in diameter (Fig. 3b, c). Images of these holes, obtained by scanning electron microscopy (SEM), reveal that they have smooth edges and do not show clear evidence of brittle rupture, suggesting that they formed when the pyroclast was above the glass transition temperature, ${T}_{{{{{\rm{g}}}}}}$. Additionally, on some clasts, larger ($\gtrsim$ 1 mm) surface ruptures are observed revealing a second skin underneath (Fig. 3d). Further photographs of individual pyroclasts can be found in Fig. S2.

### Inflated pyroclast petrography

**Fig. 4:**
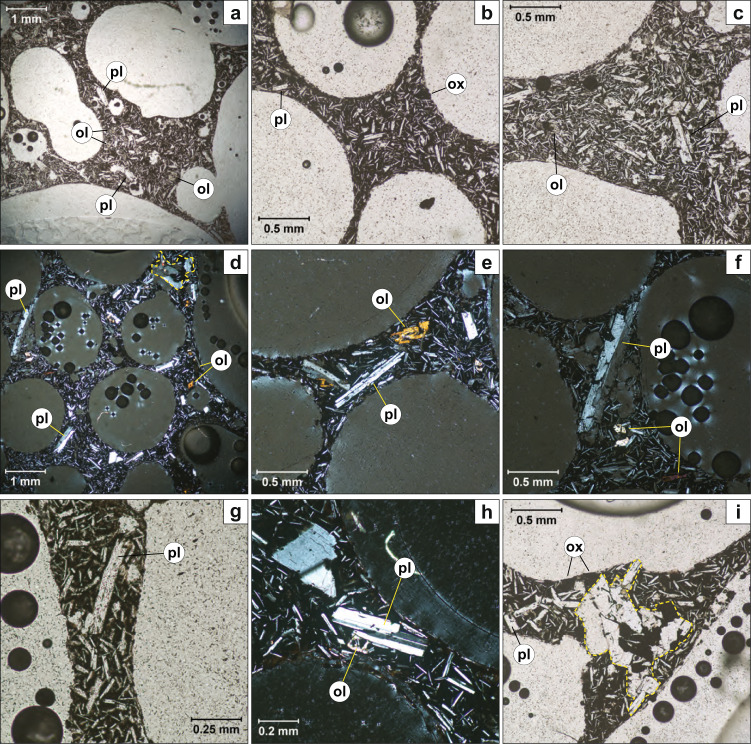
Microphotographs of an inflated pyroclast (Sample: IP_211015_A; see Fig. S2h). **a**–**c** PPL, phenocrysts of plagioclase, olivine and oxide in a microcrystalline matrix (plagioclase+olivine+oxide+ minor interstitial glass). **d**, **e** XPL, same as **a**-**c**. **f** XPL, a 1 mm long plagioclase phenocryst surrounded by three large vesicles and several smaller ones (0.1–0.2 mm in diameter). **g** PPL, close up of a thin boundary between two large vesicles. Note the dense population of plagioclase microlites. **h** XPL, a plagioclase microphenocryst and microcrystalline matrix between two vesicles. A broken fragment of a plagioclase xenocryst is in the upper left corner of the microphotograph. **i** A glomerocryst surrounded by three large vesicles. Glomerocrysts of plagioclase+olivine+oxide are outlined in a dashed yellow line in (**d**, **i**). Phenocryst phases are labelled. Abbreviations: pl: plagioclase; ol: olivine; ox: oxide (previously identified as titanomagnetite^53^).

The Tseax volcanic deposits are weakly porphyritic containing phenocrysts, glomerocrysts (>0.5 mm) and microphenocrysts (0.2–0.5 mm) of plagioclase, olivine and titanomagnetite. The dominant phenocryst phase is plagioclase (70%) followed by olivine (25%) and oxide (>5%). Clinopyroxene is never observed as a phenocryst phase. For the inflated pyroclasts, the focus of this study, we used thin section observations to record the internal texture and petrography of the pyroclast (Fig. 4). The oxides have previously been identified as titanomagnetite^53^. Plagioclase crystals show albite and polysynthetic twinning and a composition of ~An35 is obtained using the Michel–Levy Method. One large and fractured (~ 1 mm long) plagioclase is present (Fig. 4f) as well as a fragment of a plagioclase xenocryst (Fig. 4h). The olivine crystals are euhedral to subhedral, have sizes up to 0.45 mm in length, frequently exhibit hopper textures (Fig. 4e) and intracrystalline fractures are rarely filled with iddingsite. Titanomagnetite occurs as rare subhedral microphenocrysts. The sample is hyper-vesiculated (~80% vesicles), with vesicles that range in size from 0.1 mm to as large as the thin section slide (~40 mm).

The glomerocrysts consist of small aggregates of 5–12 phenocrysts of plagioclase (dominant) + olivine + oxide (Fig. 4d, i). Glomerocrysts exhibit poikilitic olivine with oxide oikocrysts and the plagioclase is interstitial to olivine and plagioclase. This suggests the following crystallisation sequence: (1) oxide, (2) olivine and (3) plagioclase. The matrix is highly crystallised and mainly composed of plagioclase microlites, olivine and oxides. Note that there is a large plagioclase microlite population even on the thinnest part of the sample (Fig. 4g). Interstitial glass is rare, and the plagioclase laths are randomly oriented suggesting that bubble growth occurred in the absence of these microlites. Only phenocrysts occasionally distort bubble shapes (Fig. 4i) and thus were present in the melt during the dominant period of bubble growth and coalescence.

### Pyroclast chemical and physical properties

All Tseax eruption products are alkali, Fe-Ti-rich basanite-to-trachybasalt in composition^53^. Bulk major element chemical compositions determined by X-Ray fluorescence measurements of representative samples are shown in Table S1.

**Fig. 5:**
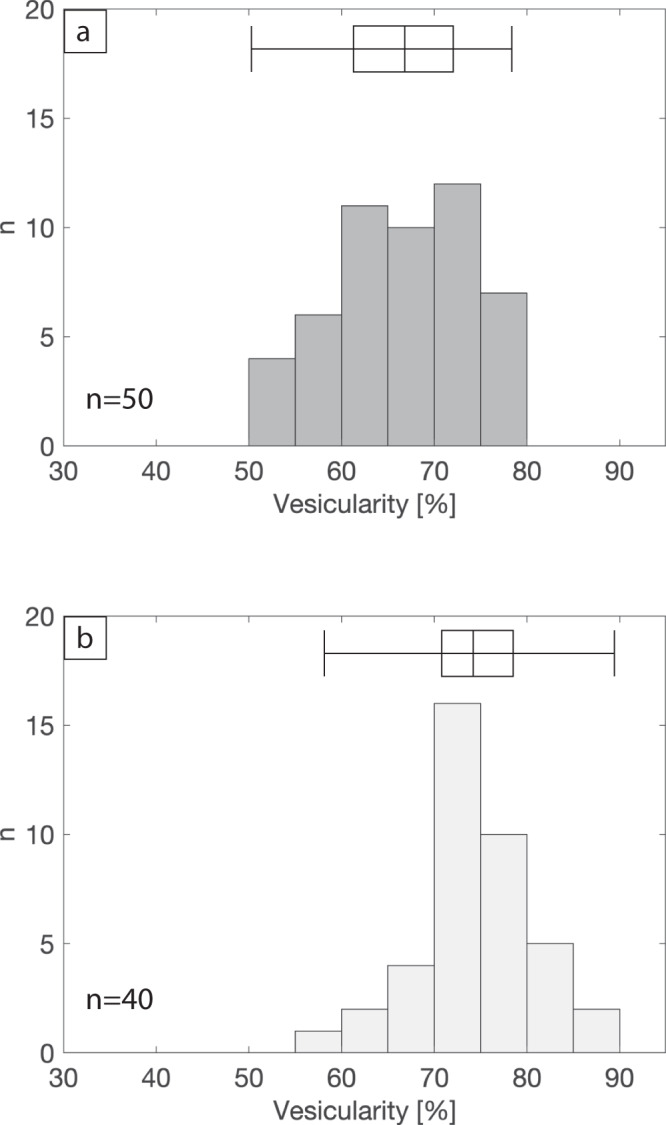
Bulk vesicularity histograms. Data shown for **a** the proximal scoria and **b** the inflated pyroclasts. The number of samples (n) measured is reported for each dataset. The box and whisker plots display the data range (whiskers) and the first quartile, median and third quartile (box). The corresponding bulk vesicularity can be found in the Supplementary Data 1 file.

Due to agglutination within the proximal fallout deposit, it was not possible to measure a full pyroclast size distribution. The axis dimensions of the five largest and five smallest inflated pyroclasts can be found in Table S2. Bulk density (i.e., combined density of rock and vesicles comprising the entire pyroclast) was measured using the Archimedes method (see Methods) on the proximal scoria and the inflated pyroclasts that could be easily removed from the deposit (Fig. 5; Supplementary Data 1). Scoria bulk density varied between 621 and 1429 kg m^−3^ with a mean bulk density of 967 kg m^−3^. Inflated pyroclast bulk density varied between 311 and 1235 kg m^−3^ with a mean bulk density of 755 kg m^−3^. Using Helium pycnometry, the dense rock equivalent of the pyroclasts was calculated to be 2957 kg m^−3^ (Fig. S3). This DRE density was used to calculate the bulk vesicularity distributions shown in Fig. 5. The scoria has a bulk vesicularity ranging from 50% to 78% with a mean of 66% and the measured inflated pyroclasts range in vesicularity from 58% to 89% with a mean of 74%.

**Fig. 6:**
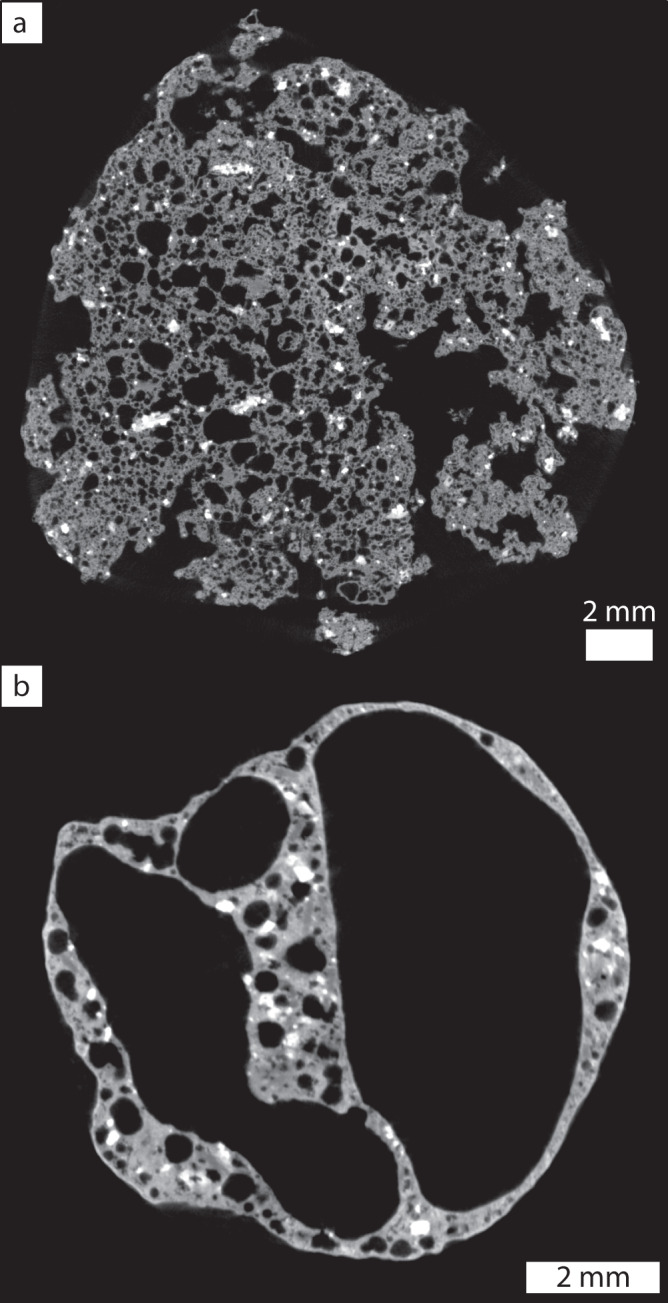
X-ray computed tomography derived images. **a** Image slice through regular scoria found adjacent to the inflated pyroclasts within the same deposit. This scoria clast has a bulk vesicularity of 76.5%. **b** Image slice through an inflated pyroclast. This inflated pyroclast has a bulk vesicularity of 89.7%. Note the difference in scale bar length between the two images.

To document the internal textures of these inflated pyroclasts and to assess how they differ from the adjacent regular scoria, a sample containing both pyroclast types was scanned using micro-X-Ray computed tomography (XRCT). Representative image slices are shown in Fig. 6a and b for the scoria and inflated pyroclast, respectively. The proximal scoria clast (Fig. 6a) has a high vesicle number density, with most vesicles sub-mm in size. The vesicles (i.e., empty pore space), shown in black, range from spherical to highly irregular in shape. Dense, crystalline phases are shown in white. (Micro)phenocrysts in the scoria forming the satellite cone contribute a total modal abundance of <16% with typical mineral abundances of 3% olivine, 3–5% plagioclase and 1–3% oxides. In the sample shown in Fig. 6a, the small ~100 μm equant crystals are interpreted to be iron oxides and the larger ~1 mm elongate laths, plagioclase. The inflated pyroclast (Fig. 6b) has a much lower bubble number density and a coarser bubble size distribution. The inflated pyroclast is dominated volumetrically by large round bubbles at its centre. These larger bubbles are enclosed by a thin $\gtrsim$50 μm crystal-bearing quenched melt film. The bubbles within this film are often rounded in shape and sometimes intersect the pyroclast exterior. In rare instances, the bubbles in the film are observed to intersect both the large internal bubble and the exterior surface of the pyroclast to form a narrowly interconnected gas-escape pathway (cf. Fig. 3b, c).

### Melt viscosity

**Fig. 7:**
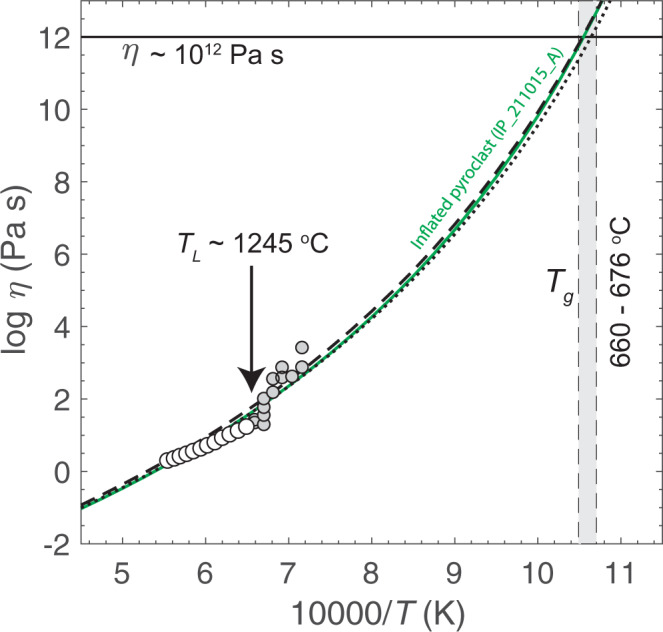
Arrhenius plot describing the viscosity, *η* (Pa s)–temperature, *T* (K) relationship for natural and remelted samples of Tseax lava. Experimentally measured values of melt viscosity (white symbols) above liquidus temperatures (*T*_*L*_ ~1245 °C) and the viscosity of melt-crystal mixtures (grey symbols). The GRD model^54^ values of melt viscosity for the composition of the natural inflated pyroclast are shown by the green labelled solid line (Table S1). Also shown are model viscosity curves for the remelted glass sample (TS-S71) predicted by the GRD model (thick dashed line) and the Raman-based model^55,56^ (fine dashed line). Model values of *T*_g_ taken as the temperature corresponding to ~ 10^12^ Pa s vary between 660 and 676 °C (grey shaded field). The corresponding rheometry data can be found in Table 1.

We measured the high-temperature, anhydrous viscosity of the Tseax melt (Table 1; Fig. 7). As detailed in Methods, the starting materials were obtained by direct fusion of the whole rock sample TS-S71 (Table S1) and were measured using a concentric cylinder apparatus in equilibrium with air at 1 atmosphere. The superliquidus anhydrous liquid viscosities ranged from 10^0.3^–10^1.2^ Pa s over the explored temperature range of 1267–1531 °C. We also measured the 1 atmosphere subliquidus viscosity where it rose to between 10^1.3^–10^3.4^ Pa s over the temperature range 1123–1243 °C. Micro-Raman spectroscopy was performed on the experimental glass to check for the presence of nanolites that may have formed during the quench; the absence of nanolites confirms that superliquidus viscosity measurements represent melt viscosities.

**Table 1:** Experimental measurements of viscosity of Tseax lava based on remelt of sample TS-S71 (see Table S1) compared to values predicted by VFT models (i.e., A_VFT_, B_VFT_, C_VFT_) for temperature dependence of melt viscosity.

State	Experimental measurements				Predicted log *η* (Pa s)	
	T (^o^C)	10,000/T(K)	log *η* (Pa s)		GRD^a^	*R*_Global_^b^
Superliquidus melt	1531	5.54	0.302		0.32	0.21
	1508	5.62	0.361		0.42	0.31
	1484	5.69	0.423		0.52	0.41
	1460	5.77	0.488		0.62	0.51
	1435	5.85	0.561		0.74	0.62
	1412	5.94	0.640		0.85	0.74
	1388	6.02	0.722		0.97	0.85
	1363	6.11	0.810		1.10	0.98
	1340	6.20	0.932		1.23	1.11
	1315	6.29	1.03		1.37	1.24
	1292	6.39	1.13		1.52	1.39
	1268	6.49	1.24		1.67	1.54
Subliquidus melt (with crystallisation)	1244	6.59	1.35	A_VFT_	−4.55	−4.55
	1244	6.59	1.43	B_VFT_	5905	5785
	1220	6.70	1.30	C_VFT_	592	590
	1220	6.70	1.56	*T*_g_ (°C)	676	667
	1220	6.70	1.78	Fragility	44.0	44.5
	1220	6.70	2.01			
	1196	6.81	2.19			
	1196	6.81	2.56			
	1172	6.92	2.88			
	1172	6.92	2.60			
	1148	7.04	2.63			
	1124	7.16	2.88			
	1124	7.16	3.42			

Raman spectrum of remelted Tseax glass (TS-S71) has a normalised peak ratio value of 0.1595.

^a^Model values predicted by GRD^54^ model.

^b^Values predicted by Raman-based model^55,56^ for melt viscosity.

Superliquidus measurements of viscosity (white circles; Fig. 7) define a smooth trend against reciprocal temperature, whilst subliquidus values of viscosity (grey circles; Fig. 7) show a sharp rise in viscosity due to crystallisation and show greater variance. The experimental dataset can be compared to model temperature-dependent curves predicted from the compositions of the remelted Tseax samples. The two sets of viscosity model parameters (GRD^54^ and Raman^55,56^) for the remelted glass are reported in Table 1 and reproduce the experimental measurements of melt viscosity well (± 0.25 log units; dashed lines Fig. 7). Furthermore, we compared these datasets to the GRD model for natural inflated pyroclast (green line; Fig. 7) compositions. Again, the near uniform chemical composition between the products yields no significant deviations in viscosity. These model curves allow for extrapolation to lower temperatures approaching the glass transition temperature (*T*_g_). The predicted values of *T*_g_ corresponding to the temperature where $\eta$ ~ 10^12^ Pa s vary between 660 °C and 676 °C below which crystallisation cannot occur.

### Origins of inflated pyroclasts

The proximal deposits at Tseax feature an intriguing juxtaposition of scoria and inflated pyroclasts that is not easily ascribed to a standard model for a thermally insulated lava fountain. In this standard model, temperature variations are predominantly controlled by the amount of entrained ambient air. A cooler fountain exterior, with a large amount of entrained ambient air, envelopes a hotter, thermally insulated, incandescent fountain interior where >*T*_g_ processes can occur^57–59^. In this conventional scenario, pyroclasts exiting the fountain cool rapidly as they pass through the cooler exterior; the extent of cooling being dictated by their size and trajectory^30,32,40,58^. The large pyroclasts (e.g., spatter) may exit the fountain hot (>*T*_g_) allowing dynamic processes to continue modifying their interiors (i.e., coalescence, vesiculation)^12,13^. Smaller pyroclasts tend to be cooled to below *T*_g_ except for those with very short trajectories as found in very proximal deposits.

In the highly proximal tephra deposits, the inflated pyroclasts represent a substantially smaller volume fraction of the deposit than does scoria. However, the inflated pyroclasts are found over a wide range of sizes (Fig. 2). They are not restricted to the largest sized pyroclasts but can be found as small as ~0.2 cm (post inflation). Furthermore, there is clear macro- and microscale evidence for the bubble growth, which drives the inflation, to occur within the deposit, after deposition. Thus, the inflated pyroclasts can only occur under one of two distinct eruption scenarios: (1) extreme thermal heterogeneity within the lava fountain or (2) abrupt transitions from lava fountaining to discrete bubble bursts. These two hypotheses will now be evaluated for the Tseax eruption.

**Fig. 8:**
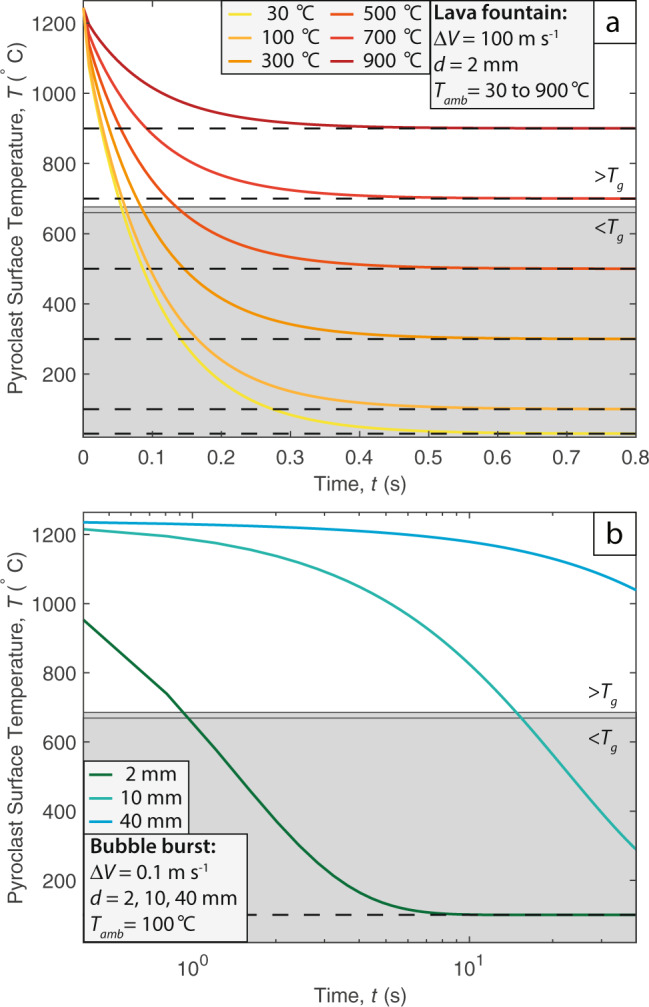
Heat conduction model for pyroclasts. The coloured lines track the surface temperature of the pyroclast. The black dashed lines represent the corresponding ambient air temperature, and the grey shaded region denotes the temperatures below the glass transition temperature, *T*_g_. Both *T*_g_ values of 660 °C and 676 °C are marked to represent the range determined by high-temperature rheometry. **a** Cooling of a 2 mm diameter pyroclast within a lava fountain, the ambient temperatures ranging from 30°C (light yellow) to 900 °C (dark red) represent the thermal zonation in the fountain, from the cold edge to the hotter, insulated interior. **b** Cooling of 2 mm (dark green), 10 mm (light green) and 40 mm (blue) diameter pyroclasts generated from a low-velocity bubble burst. Note, the change of the x-axis to a log scale.

Extreme thermal heterogeneities within the parent lava fountain could account for the contrasting thermal histories of the scoria and inflated pyroclasts, where a small volume of thermally insulated lava proceeds to form the inflated pyroclasts. To assess the feasibility of this scenario at Tseax, we used a 1D transient heat conduction model for a sphere^30,60^ (see Methods). The observed field deposits require that all inflated pyroclasts, irrespective of their size, landed and inflated in the deposit above *T*_g_. Furthermore, no breadcrust textured surfaces were observed indicating that the entire inflated pyroclast, including its surface was kept well above *T*_*g*_ during transport and emplacement. Given that pyroclasts loose heat as function of their size, we modelled the cooling of a pyroclast 2 mm in diameter exposed to different ambient temperatures (Fig. 8a). This small particle diameter is consistent with the smallest inflated pyroclasts observed in the deposit (Table S2) and therefore provides the most robust hypothesis test. As shown in Fig. 8a, pyroclasts are only able to stay hot enough to form inflated pyroclasts when the ambient temperature is above the glass transition temperature. When the ambient temperature drops below the glass transition temperature (cooler lines; Fig. 8a), the pyroclast becomes effectively solid in <0.2 s and therefore cannot inflate in the deposit.

Thus, for a standard thermally insulated lava fountain^30,61,62^, only pyroclasts that reside in the hot ($\gtrsim 700^\circ$C) interior for the entirety of the transport path can form inflated pyroclasts. For the modelled conditions at Tseax this is highly unlikely; firstly, a 2 mm pyroclast would not be able to settle vertically within an opposing gas fountain velocity of ~100 m s^−1^. Secondly, pyroclasts are likely to enter the cooler exterior either at the fountain top or edges, from this point transport and in-deposit inflation must occur on timescales < 0.2 s, this is infeasible. However, if ambient gas temperatures surrounding the fountain are locally raised to temperatures above *T*_*g*_ for short periods of time, pyroclasts could exit the fountain and reach the deposit above *T*_*g*_, potentially forming inflated pyroclasts. Such high gas temperatures ($\gtrsim 700^\circ$C) surrounding lava fountains are unusual, although they have been documented^33^ during the 2018 eruption of Kīlauea and are most likely when mass eruption rates are high, the vent is wide, and hosts ponded lava. We therefore cannot rule out the production of inflated pyroclasts via extreme thermal anomalies at other localities, but suggest that it is highly unlikely at Tseax.

Hawaiian style fountains are inherently unsteady and during a single eruption episode the fountain height can vary substantially^18^. These intra-episode height variations are often due to changes in the mass eruption rate^40,58^ but other factors such as proximal lava ponding around the vent can also play a role^34,58^. Inflated pyroclasts may form instead of scoria if the lava fountain temporarily pauses or stops and the eruptive activity transitions to low-energy bubble bursts^63^ (similar to bubble bursting at a lava lake or very weak Strombolian activity). Such eruptive behaviour transitions are not unprecedented and similar abrupt (but regular) changes in eruptive activity have been witnessed during the 2021 eruption of Fagradalsfjall, Iceland and during the 2018 eruption of Kīlauea, Hawaii^21^. The cause of such transitions remains uncertain, but it is likely due to reorganisation of gas in the shallow volcanic plumbing system. This forms our second hypothesis and is again tested through thermal modelling (Fig. 8b).

**Fig. 9:**
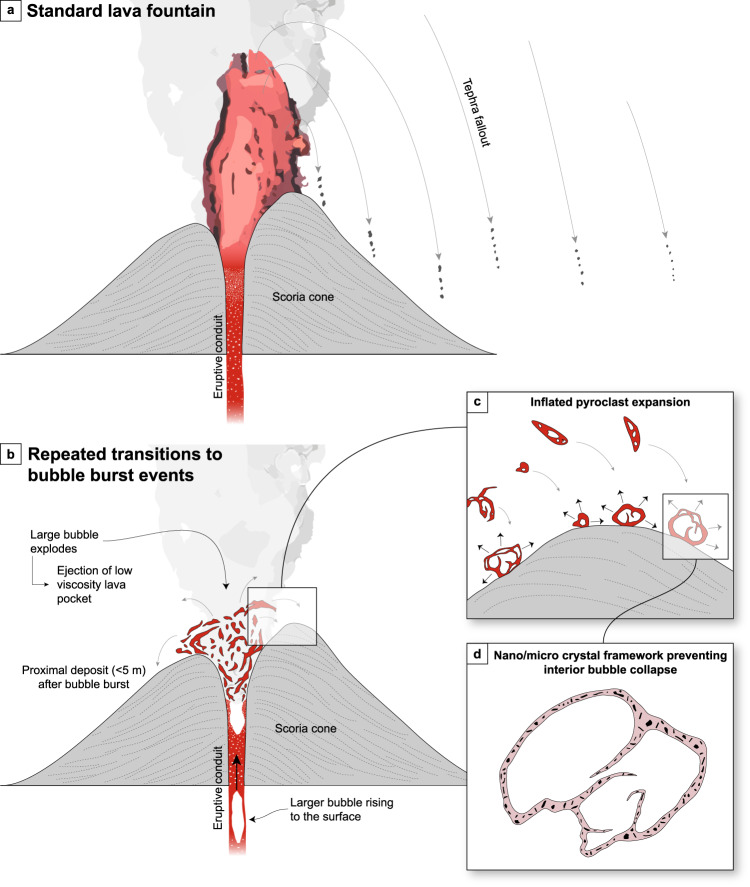
Summary cartoon of the eruptive processes that operated at the Satellite cone, Tseax volcano. The diagrams are schematically drawn and are not to scale. **a** Standard lava fountain producing regular scoria. **b** Repeated transitions to a low-energy bubble burst ejecting low viscosity lava fragments that land in close proximity to the vent. **c** Close up of the proximal deposits showing the interior bubble growth and in-deposit inflation of the pyroclasts. **d** Sketch of a cross-section through an inflated pyroclast with a crystal framework preventing interior bubble collapse.

During low-energy bubble bursts, the differential velocity between the ejected clast and the surrounding air is low compared to lava fountain events. Lower differential velocities correspond to lower heat transfer coefficients meaning that cooling is less effective and pyroclasts can remain hot, and above the glass transition temperature, for longer. These conditions are shown in Fig. 8b, where a 2 mm diameter pyroclast (including the exterior surface) remains above *T*_g_ for ~1 s: an order of magnitude longer than the lava fountain scenario (cf. Fig. 8a). Furthermore, if the full range of inflated pyroclast sizes are considered (sequential coloured lines in Fig. 8b), we show that the majority of inflated pyroclasts required 10’s or 100’s of seconds to reach *T*_g_—ample time for the pyroclasts to be ejected, reach the deposit, and for the bubbles to grow and coalesce to produce the textures observed. We therefore contend that repeated rapid transitions from lava fountaining (producing the regular scoria) to discrete low-energy bubble bursts provided the in-deposit conditions required to form the inflated pyroclasts at Tseax (Fig. 9).

The melt properties also played a role in the formation of inflated pyroclasts. The Tseax magma has a low viscosity and a predicted abnormally low glass transition temperature, *T*_g_ (660–676 °C; Fig. 7). These physical properties are key to the formation of inflated pyroclasts. Firstly, the low melt viscosity allows for bubble growth and coalescence to occur on timescales faster than pyroclast cooling. Secondly, a low *T*_g_ increases Δ*T* = *T*_e_ − *T*_g_ thereby extending the time available for pyroclast modification after primary magmatic fragmentation. Thirdly, the iron-rich pyroclasts were subject to rapid cooling—conditions that favour microlite crystallisation^52^. For these melt compositions it has been shown^53^ that plagioclase and oxides will rapidly crystalize at temperatures just below the Tseax eruption temperature, *T*_e_. Supported by our thin section observations, we suggest that extensive plagioclase microlites formed towards the end of pyroclast inflation to generate the non-glassy surface texture observed (Fig. 3a). Furthermore, these microlites provided a supporting framework, and a rise in viscosity (Fig. 7), that allowed the inflated pyroclasts to retain their shape, rather than collapsing, as interior bubbles breached the exterior surface of the pyroclast allowing egress of pressurised gasses (Fig. 3b–d).

### The critical conditions required to produce inflated pyroclasts

Here, we have documented the conditions that lead to inflated pyroclasts at Tseax but contend that inflated pyroclasts could occur at a range of volcanoes worldwide, provided a series of critical conditions are met. Thus, when recognised in deposits, inflated pyroclasts indicate the following eruption conditions:

- Pyroclast velocities were small, and certainly lower than the critical impact velocity causing pyroclasts to highly deform on impact and further fragment into a series of droplets^13^.
- The eruption temperatures were hot enough and/or the flight trajectories were short enough and/or in-flight cooling was limited such that clasts land in the deposit with temperatures > *T*_g_.
- Melt viscosities were low to allow bubbles to grow, relax and coalesce on short timescales.
- The eruption/episode volume was small and the deposit accumulation rate low, below the threshold that would produce rheomorphic lava flows^14^.
- If vesicles are observed intersecting the pyroclast exterior, microlites and/or nanolites may have formed preventing interior bubble collapse.
- If scoria and inflated pyroclasts are intermixed within the deposit (rather than a single horizon of inflated pyroclasts), then transitions between fountaining and bubble bursts occurred multiple times.

We have shown that when juxtaposed with normal scoria, inflated pyroclasts are indicators of abrupt transitions in eruptive style—repeated rapid transitions between lava fountaining activity and low-energy bubble bursts— transitions that could be considered when hazard planning and managing access to these (basaltic) eruptions that are popular with tourists.

## Methods

### Deposit sampling

The samples were collected during three field seasons (Summers of 2016, 2017 and 2019) at Tseax volcano (55.11085 °N, 128.89944 °W), in northwest British Columbia, Canada. The inflated pyroclasts sampled for this study were collected within a 5 m radius of the summit of the Satellite cone (Fig. 1). No deposit stratigraphy was identified so the inflated pyroclasts were sampled from the present-day surface, or from the upper 0.5 m of deposit when an erosional surface exposed a small section. If inflated pyroclasts were too agglutinated to be successfully removed from the deposit a larger sample was taken for later, careful separation in the lab. During transport, the samples were individually wrapped in bubble wrap to prevent breakage, comminution, and surface modifications.

### Scanning electron microscopy analysis

Six representative inflated pyroclasts were cut into ~2 cm pieces and mounted on scanning electron microscope (SEM) stubs using adhesive carbon tape to reduce sample charging. All sample stubs were then carbon coated using a sputter coater. Analysis and image acquisition were then performed using a Philips XL30 SEM in scanning electron mode with a 15 kV accelerating voltage, a 35 μA beam current, and an average working distance of 11 mm.

### Electron microprobe analysis

The major element compositions of glasses derived from remelting of whole rock sample (TS-S71; see viscosity) were measured using a Cameca SX100 electron probe microanalyser (EPMA) at the Department for Earth and Environmental Sciences, LMU Munich, Germany. Operating conditions were an acceleration voltage of 15 kV and beam current of 4 nA, using a defocused 10 µm beam to minimise alkali loss. The peak time of our analyses was 10 s and a background time of 5 s (on each side of the peak) for all elements. Albite, periclase, apatite, wollastonite, bustamite, Fe_2_O_3_ and ilmenite were used as standards. Standard deviations based on replicate measurements are < 2.5% for all analysed elements. Precision and accuracy were checked by analysing the reference glasses VG-2 (basalt) and VG-568 (rhyolite)^64,65^ at the start of each analytical session. No significant alkali loss was observed relative to tabulated values of standards.

### X-Ray fluorescence

X-Ray fluorescence measurements of the bulk major element chemical compositions of representative samples were measured by ALS Minerals in North Vancouver, British Columbia (Table S1). Ferrous iron (FeO wt.%) content was measured by volumetric analysis. The detection limit is 0.01 wt.% for all the major oxides. The sample suite includes: (i) samples of highly oxidised tephra from the satellite cone (SC) where the inflated pyroclasts are found, (ii) tephra from the main cone (MC) which overlies SC tephra, (iii) a sample of lava (TS-S71), (iv) an inflated pyroclast (IP211015A) and (v) the glass produced by remelting the lava in support of the viscosity measurements.

### Dense rock equivalent, or skeletal density measurements

Using a mortar and pestle, several inflated pyroclasts were crushed to a fine powder, finer than the smallest vesicles. The dense rock equivalent, or skeletal density of the inflated pyroclasts was calculated using an analytical balance to measure mass and a Micrometrics Accupyc II 1340 helium pycnometer to measure volume. Five aliquots of the fine pyroclast powder were measured ten times for mass and volume, then plotted as mass against volume. A linear regression was then fitted through the five datasets and the origin, with the slope determining the density. These data are shown in Fig. S3.

### Bulk density measurements

A total of 40 inflated pyroclasts and 50 scoria clasts were measured for bulk density using the Archimedes techniques outlined in Houghton & Wilson^66^. The bulk vesicularity was calculated from the bulk density using the dense rock equivalent density measured independently by helium pycnometry (as detailed above). Three repeat measurements on the same inflated pyroclast revealed a maximum absolute uncertainty of ± 1% vesicularity.

### Viscosity measurements

We measured the anhydrous, 1 atmosphere viscosity of the remelted TS-S71 sample (Tables 1 and S1). Viscosity of the Tseax melt was measured using standard rheological measurements. High-temperature viscosity measurements were made, after normal calibration procedures, at the Department for Earth and Environmental Sciences, LMU Munich, Germany. A concentric cylinder apparatus was used for sample homogenisation and determination of anhydrous liquid viscosities (10^0.30^−10^3.42^ Pa s) at both superliquidus (1531–1267 °C) and subliquidus (1244–1124 °C) temperatures.

The measured liquid viscosity of the Tseax melt is one of the lowest values amongst those measured for natural samples^54^. The subliquidus measurements show that the onset of an apparent crystallisation (<1244 °C) has a measurable rheological effect manifest by a drift in viscosity values with lower temperatures. The high temperature marking the onset of crystallisation is not typical of other basaltic melts including MORB, OIB, or more alkaline, iron-poorer basalts^67^.

We used the composition of the remelted glasses of TS-S71 to calculate the temperature dependence of the liquid viscosity using the GRD model^54^. The corresponding VFT function^68–70^ is:

1 $${{\log }}\,\eta =\,-4.55+\,\frac{5904.6}{T\left(K\right)-591.8}$$

which predicts the measured values well (cf. Fig. 7).

### X-ray computed tomography

One ~4.5 cm sample containing both scoria and an adhering inflated pyroclast was scanned using a Scanco Medical µCT100 device housed at the Centre for High-Throughput Phenogenomics, Faculty of Dentistry, University of British Columbia. Acquisition conditions were a 55 kV accelerating voltage, a 200 µA current and a 0.5 mm aluminium filter. The total exposure time was 9.7 h. The voxel dimension (i.e., the resolution) is 11.4 µm.

### Thermal models

We modelled the cooling of inflated pyroclasts using a 1D transient heat conduction model based on the MATLAB code by Recktenwald^60^ as used by Porritt et al.^30^. Here the pyroclasts are modelled as spherical and lose heat from their surfaces by forced convection, ${F}_{{{{{\rm{c}}}}}}$:

2 $${F}_{{{{{\rm{c}}}}}}={h}_{{{{{\rm{c}}}}}}({T}_{{{{{\rm{s}}}}}}-{T}_{{{{{\rm{a}}}}}})$$

where ${h}_{{{{{\rm{c}}}}}}$ is the heat transfer coefficient, ${T}_{{{{{\rm{s}}}}}}$ is the surface temperature of the pyroclast and ${T}_{{{{{\rm{a}}}}}}$ is the ambient temperature. Given that small microphenocrysts are observed in the inflated pyroclasts, we set ${T}_{{{{{\rm{s}}}}}}$ to be 1244 °C, just below the liquidus temperature. Various values for ${T}_{{{{{\rm{a}}}}}}$ were used ranging from 900 °C and 30 °C which represent the core of a vigorous lava fountain and background air temperature, respectively. The heat transfer coefficient, ${h}_{{{{{\rm{c}}}}}}$ is encapsulated within the Nusselt number, Nu, a dimensionless heat loss parameter defined as:

3 $${{{{{\rm{Nu}}}}}}=\frac{{h}_{{{{{\rm{c}}}}}}d}{{k}_{{{{{\rm{a}}}}}}}$$

where $d$ is the pyroclast diameter and ${k}_{{{{{\rm{a}}}}}}$ is the thermal conductivity of the air (0.0257 Wm^−1^ K^−1^). We calculated Nu based on previous experimental work that used spheres^71^ and natural volcanic pyroclasts^72^. The equations are based on two further dimensionless groups, the particle Reynolds number, Re, and the Prandtl number, Pr. First, the particle Reynolds number is given by:

4 $${{{{{\rm{Re}}}}}}=\frac{\triangle {Vd}{\rho }_{{{{{\rm{a}}}}}}}{{\mu }_{{{{{\rm{a}}}}}}}$$

where $\triangle V$ is the differential velocity between the ejected pyroclast and the surrounding air, ${\rho }_{a}$ is the air density and ${\mu }_{{{{{\rm{a}}}}}}$ is the air viscosity. The temperature dependence of air density and viscosity is assumed to be negligible, and we use constant values of 1.225 kg m^−3^ and 1.75 × 10^−5^ Pa s, respectively. We assume the average pyroclast differential velocity, $\triangle V$ to be ~100 m s^−1^ for a lava fountain^30,73^ and ~0.1 m s^−1^ for a bubble burst event^63^ considering drag and the entire ballistic motion. Second, the Prandtl number is given by:

5 $${{{{{\rm{Pr }}}}}}=\frac{{\mu }_{{{{{\rm{a}}}}}}{C}_{{{{{\rm{p}}}}}}}{{k}_{{{{{\rm{a}}}}}}}$$

where ${C}_{{{{{\rm{p}}}}}}$ is the air heat capacity, here taken as a constant value of 1005 J kg^−1^ K^−1^.

Previous experimental work^71,72^ found that for low Reynolds numbers, $<$ 2.0 × 10^5^, the Nusselt number is given by the following relationship:

6 $${{{{{\rm{Nu}}}}}}=2+x{{{{{{\rm{Re}}}}}}}^{1/2}{{{\Pr }}}^{1/3}$$

where $x=2.2\times {10}^{-4}\rho +0.31$ and $\rho$ is the density of the pyroclast, taken here to be the mean bulk density of the proximal scoria (967 kg m^−3^; Fig. 5). The largest particle Reynolds number used in Fig. 8 was 1.4 × 10^4^, thus Eq. 6 is valid. The cooling model also uses the melt thermal conductivity and diffusivity; here we assume these to be constant and use typical values of 2 W m^−1^ K^−1^ and 1 × 10^−5^ m^2^ s^−1^, respectively^74–78^.

## Supplementary information

Supplementary Information

Description of Supplementary Material

Supplementary Dataset 1

## Data Availability

The rheometry, bulk density, geochemical and field data generated or analysed in this study are provided in the main article, the Supplementary Information, and Supplementary Data 1. The original X-Ray Computed Tomography data are available from the authors on request.

## Code availability

The 1D transient heat conduction code is written in MATLAB and adapted from Recktenwald^60^. Our adapted version can be accessed via Github/Zenodo (10.5281/zenodo.6381629).
