# Supplementary material for: Inflated pyroclasts in proximal fallout deposits reveal abrupt transitions in eruption behaviour
Source: Nat Commun. 2022 May 20;13:2832. doi: 10.1038/s41467-022-30501-6 (PMC9122929; doi:10.1038/s41467-022-30501-6)
Supplement: Supplementary file 1 — Supplementary Information [file 41467_2022_30501_MOESM1_ESM.pdf]

Supplementary Information to Accompany:

Inflated pyroclasts in proximal fallout deposits reveal abrupt transitions in eruption behaviour

Thomas J. Jones, Yannick Le Moigne, James K. Russell, Glyn Williams-Jones, Daniele Giordano, Donald B. Dingwell

Included in this pdf file:

Table S1
Description: Major element chemical compositions (wt.%) of Tseax samples.

Table S2
Description: Long, intermediate, and short axis of the five smallest and largest inflated pyroclasts observed at Tseax.

Figure S1
Description: Additional field photographs showing the inflated pyroclasts within the proximal deposit at the Satellite cone, Tseax volcano.

Figure S2
Description: Photographs of a range of individual inflated pyroclasts collected in the field.

Figure S3
Description: Dense rock equivalent (DRE) density of the inflated pyroclasts.

Table S1. Major element chemical compositions (wt.%) of Tseax pyroclasts (MC, Main cone, SC Satellite cone), and remelted glasses and calculated properties including: VFT coefficients for viscosity and ancillary properties based on Giordano et al.[54] Data sources are shown by the following superscript characters: [a]Le Moigne et al.[44]; [b]R.Gallo BSc thesis; [c]Mean electron microprobe analysis (N= 10, ±1s); [d]Tg is taken as the temperature where melt viscosity ~10^{12} Pa s; [e]Fragility, *m* see text)

Sample<br>Description<br>Volcano	TS-S40A[a]<br>tephra<br>SC (Base)	TS-S40B[a]<br>tephra<br>SC (Base)	RG-S22a[b]<br>tephra<br>SC (Flank)	RG-22b[b]<br>tephra<br>MC	TS-S71[c]<br>glass<br>Remelt	IP_211015_A<br>inflated pyroclast<br>SC (Near vent)
SiO_2	45.20	44.90	46.10	46.00	48.09 (0.25)	46.09
TiO_2	3.46	3.45	3.63	3.56	3.56 (0.11)	3.66
Al_2O_3	14.25	14.20	14.45	14.51	14.42 (0.17)	14.50
Fe_2O_3	15.05	14.81	14.24	5.01	-	12.23
FeO	1.44	1.70	2.56	10.85	14.31 (0.36)	3.70
MnO	0.22	0.22	0.22	0.22	0.17 (0.08)	0.22
MgO	4.24	4.21	4.38	4.26	4.20 (0.12)	4.44
CaO	6.67	6.59	6.88	7.30	7.80 (0.15)	7.54
Na_2O	4.05	4.12	3.76	3.18	4.04 (0.17)	3.89
K_2O	1.86	1.87	1.76	1.77	1.75 (0.07)	1.77
P_2O_5	1.12	1.12	1.14	1.18	1.11 (0.14)	1.11
H_2O^+	-	-	-	-	-	0.31
SO_3	-	-	0.21	0.15	-	0.05
CO_2	-	-	0.11	0.66	-	0.37
**Total**	97.56	97.19	99.44	98.65	99.45	99.87
Fe_2O_3 (T)	16.65	16.7	17.09	17.07	15.9	16.34
LOI	0.74	0.66	-0.02	-0.51	-	0.04
Mg#	33.53	33.31	33.68	33.09	34.35	34.99
**Model VFT functions and Calculated Properties**						
A	-4.55	-4.55	-4.55	-4.55	-4.55	-4.55
B	5730.7	5718.8	5716.2	5688.9	5904.6	5702.8
C	596.9	596.7	601.8	608.3	591.8	603.1
Tg (K)[d]	943	942	947	952	949	948
Fragility[e]	45.1	45.1	45.4	45.8	44	45.5

Table S2. Long (a), intermediate (b), and short (c) axis of the five smallest and largest inflated pyroclasts observed at Tseax.

**Smallest**			**Largest**		
**a (mm)**	**b (mm)**	**c (mm)**	**a (mm)**	**b (mm)**	**c (mm)**
1.89	1.44	1.30	49.06	31.75	30.83
2.51	2.08	1.65	46.96	29.56	20.67
2.03	1.38	1.33	41.35	36.67	20.36
3.17	2.05	1.31	40.20	37.40	26.13
2.17	2.01	1.89	35.87	32.74	9.04

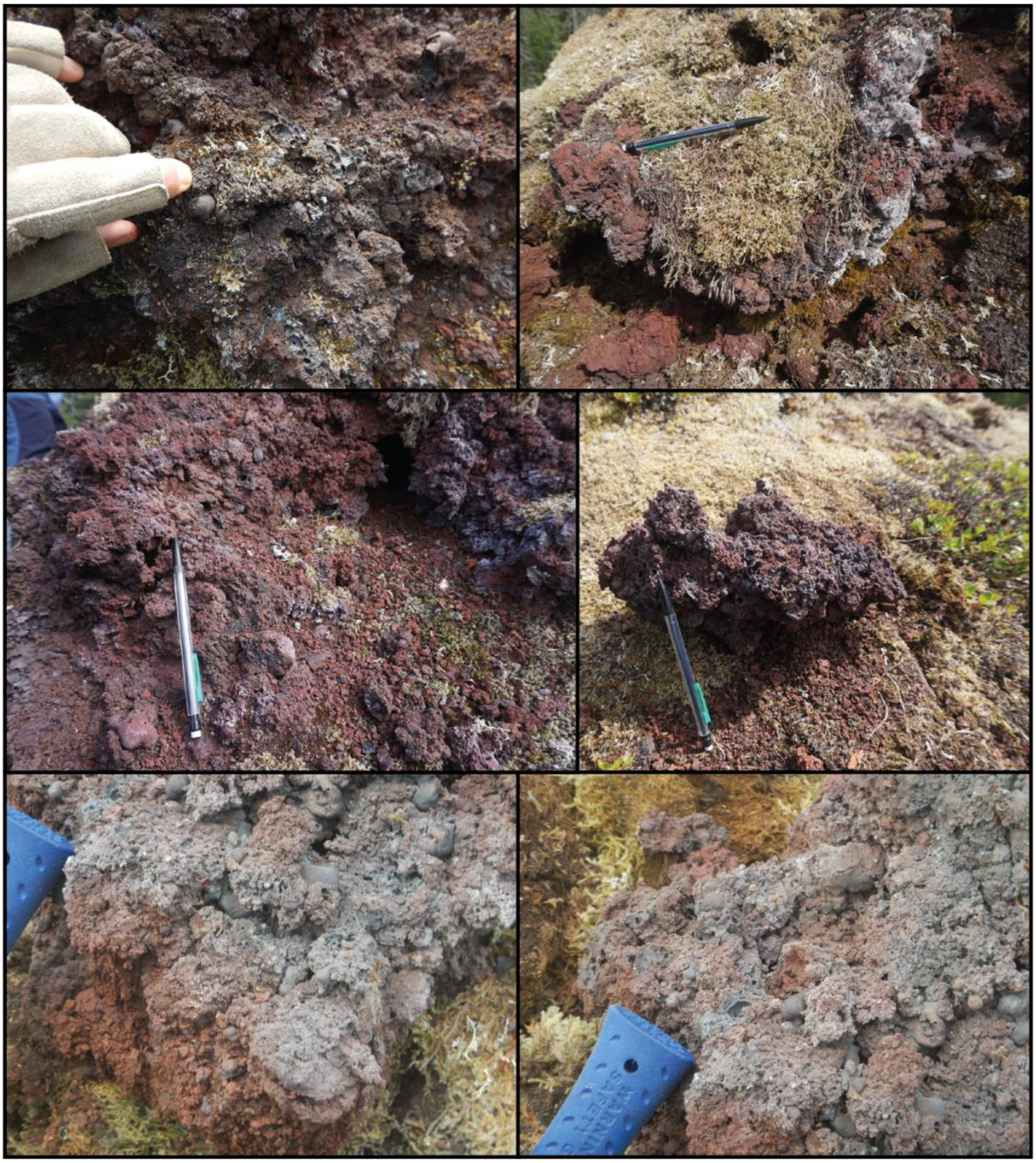

Figure S1. Additional field photographs showing the inflated pyroclasts within the proximal deposit at the Satellite cone, Tseax volcano.

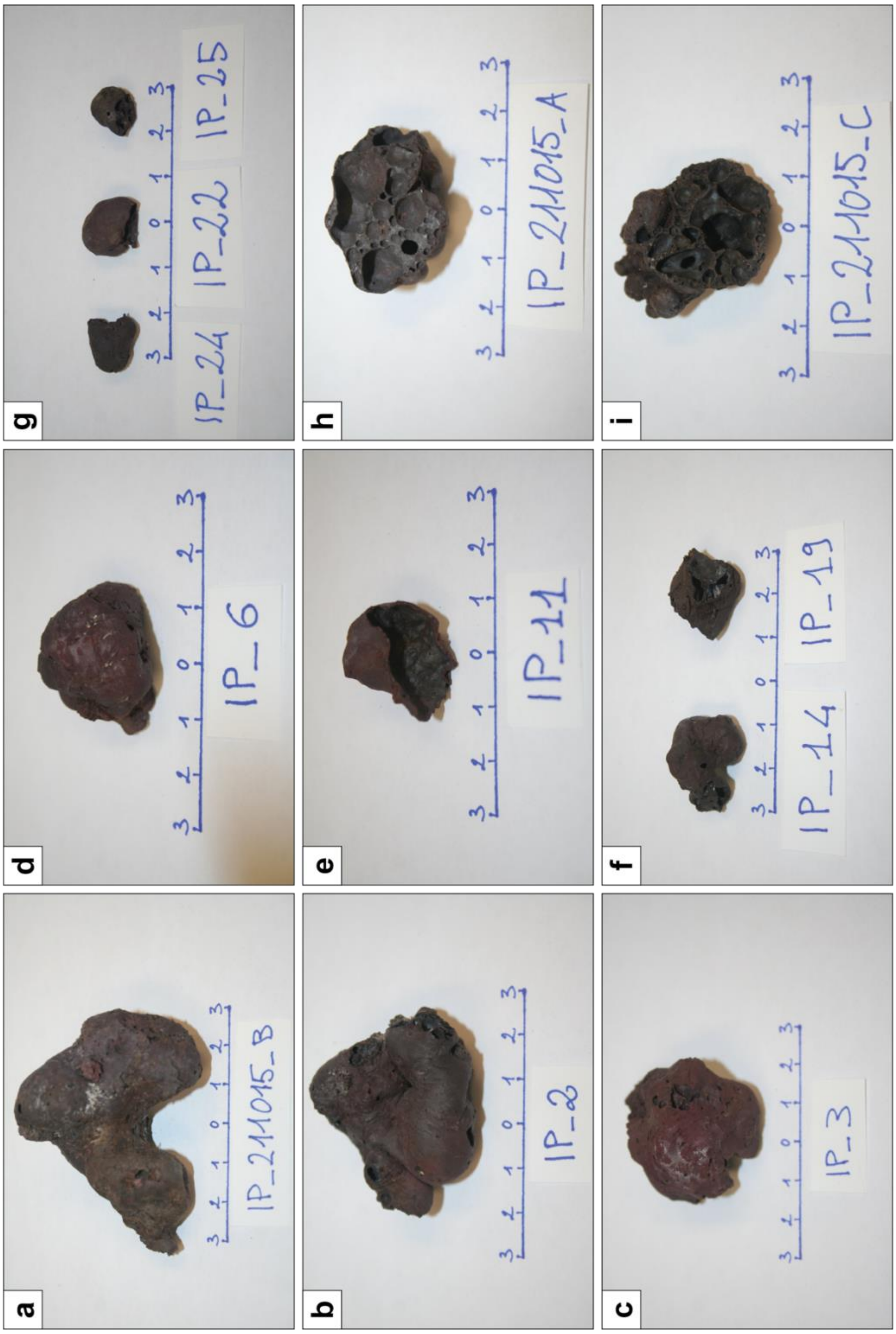

Figure S2. Photographs of a range of individual inflated pyroclasts collected in the field. The scale bar is in cm and the text is the sample label. Different pyroclast sizes are shown as follows: **(a)** 6.0 x 3.9 x 3.1 cm; **(b)** 4 x 3.7 x 2.6 cm; **(c)** 4.1 x 3.7 x 2.0 cm; **(d)** 2.8 x 2.1 x 1.9 cm; **(e)** broken specimen, 2.2 x 1.7 x 1.4 cm; **(f)** two specimens: IP_14 is 1.9 x 1.7 x 1.1 cm, IP_19 is 1.6 x 1.5 x 1.5 cm; **(g)** 3 specimens: IP_22 is 1.2 x 1.1 x 0.9 cm, IP_24 is 1.2 x 0.9 x 0.8 cm, IP_25 is 0.9 x 0.8 x 0.8 cm. **(h & i)**: broken specimens showing their hyper-vesiculated interior.

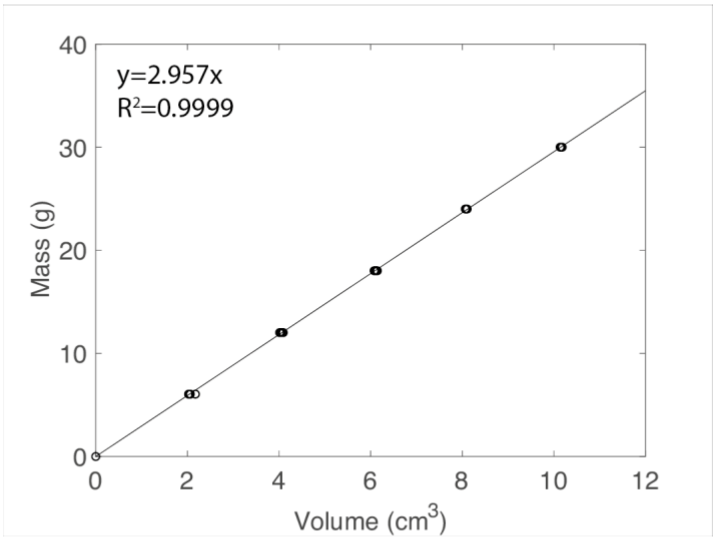

Figure S3. Helium pycnometry data for finely crushed inflated pyroclasts plotted as a function of volume (cm^3) and mass (g). Each mass has ten volume measurements. Linear regression (R^2 = 0.9999) yields a dense rock equivalent (DRE) density of 2.957 g cm^{-3} with lower and upper 95 % confidence bounds of 2.953 g cm^{-3} and 2.960 g cm^{-3}, respectively.
