# Supplementary material for: Inflated pyroclasts in proximal fallout deposits reveal abrupt transitions in eruption behaviour
Source: Nat Commun. 2022 May 20;13:2832. doi: 10.1038/s41467-022-30501-6 (PMC9122929; doi:10.1038/s41467-022-30501-6)
Supplement: Supplementary file 2 — Description of Supplementary Material [file 41467_2022_30501_MOESM2_ESM.pdf]

Description of Additional Supplementary Files

File name: Supplementary Data 1.xlsx
Description: An excel file containing all the raw bulk vesicularity data plotted in Figure 5. The file has two tabs, corresponding to the data plotted in the two figure panels a and b.
